# Acquired partial lipodystrophy (Barraquer-Simons syndrome) with both-side axillary breasts: A case report

**DOI:** 10.1016/j.ijscr.2025.111202

**Published:** 2025-03-25

**Authors:** Mohammad Al-Jawad, Lina Mawaldi, Nada Mawaldi, Mariam Krimsty, Silva Ishkanian

**Affiliations:** University of Aleppo, Faculty of Medicine, Aleppo, Syria

**Keywords:** Lipodystrophies, Partial lipodystrophies, Case report

## Abstract

**Introduction:**

Lipodystrophies are conditions characterized by a loss of subcutaneous fat, which can be congenital or acquired, and present in various patterns of fat distribution. Accurate diagnosis is essential for identifying comorbidities and ensuring appropriate multidisciplinary care and targeted treatments.

**Case presentation:**

A 34-year-old female experienced significant facial changes and increased abdominal fat over five years. She had lumps under her armpits discharging white fluid, with clinical findings of low hemoglobin and accessory breast tissue, which was surgically excised.

**Discussion:**

Acquired generalized lipodystrophy is often overlooked, with over 250 documented cases. It typically presents in adolescence and involves symmetrical fat loss, with potential associations to autoimmune diseases and unique features in this case.

**Conclusion:**

This case emphasizes the complexities of acquired lipodystrophy, especially regarding accessory breast tissue. It highlights the need for thorough evaluations and increased awareness among healthcare providers for timely diagnosis and management.

## Highlights

• A 34-year-old woman presented with significant facial changes and abnormal fat distribution, indicative of acquired generalized lipodystrophy.
• She also exhibited accessory breast tissue.
• This case emphasizes timely diagnosis of acquired lipodystrophy for better patient outcomes.

## 1 Introduction

Lipodystrophy is a condition marked by the complete or partial loss of adipose (fat) tissue, as well as abnormal distribution of fat in specific areas of the body [1].

Lipodystrophy (LS) can be either primary or acquired and manifests in various clinical forms. It is typically classified according to the distribution of fat loss, ranging from partial to generalized forms of LS. Partial LS is likely underdiagnosed. Accurate diagnosis of lipodystrophy is crucial, as it necessitates the identification of specific comorbidities, especially cardiac issues, and supports the need for multidisciplinary care, which may include the initiation of targeted treatments in certain cases [2].

It can manifest at any age, ranging from early infancy to adulthood, with onset typically occurring in the first or second decade of life. The prognosis is generally favorable, though mortality and morbidity are influenced by the involvement of associated organ systems and any comorbid conditions present [3].

In our case we present a case of 34-year-old which diagnosed to have acquired partial lipodystrophy with both-side axillary breasts that was treated successfully, and we highlight the challenges encountered in managing this unique case as per the SCARE checklist [4].

## 2 Case presentation

**Fig. 1:**
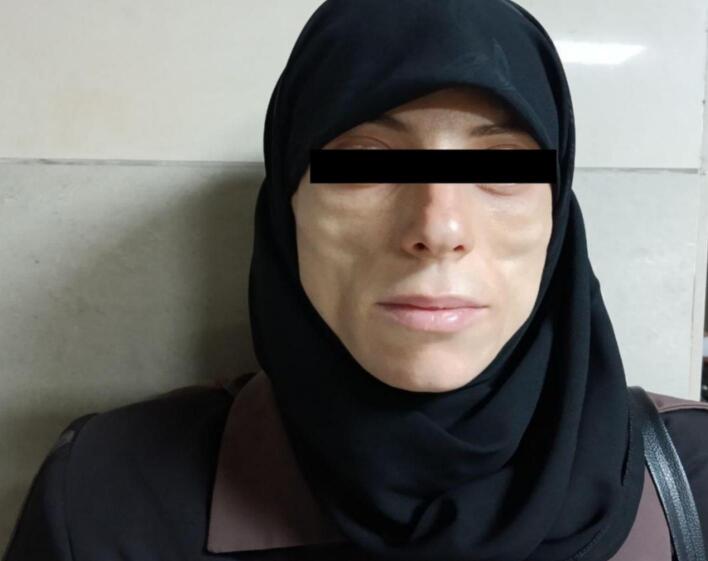
A 34-year-old woman with significant facial changes and worsening wrinkles, leading to a loss of self-recognition over five years.

**Fig. 2:**
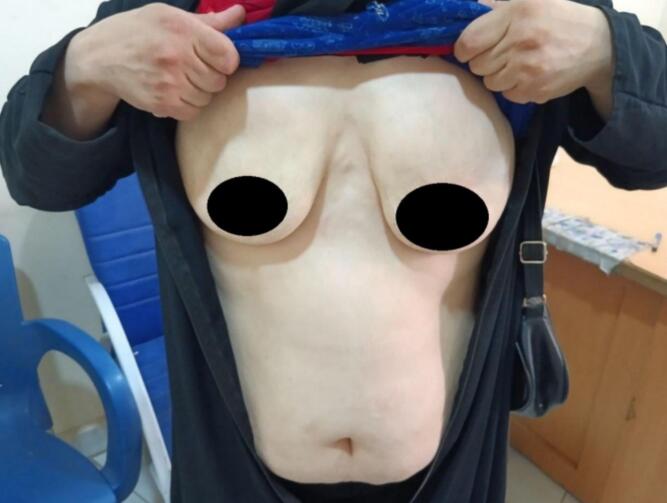
increased adipose tissue in the abdominal and lower limb areas, alongside a noticeable reduction in fat in the upper body (chest and arms).

**Fig. 3:**
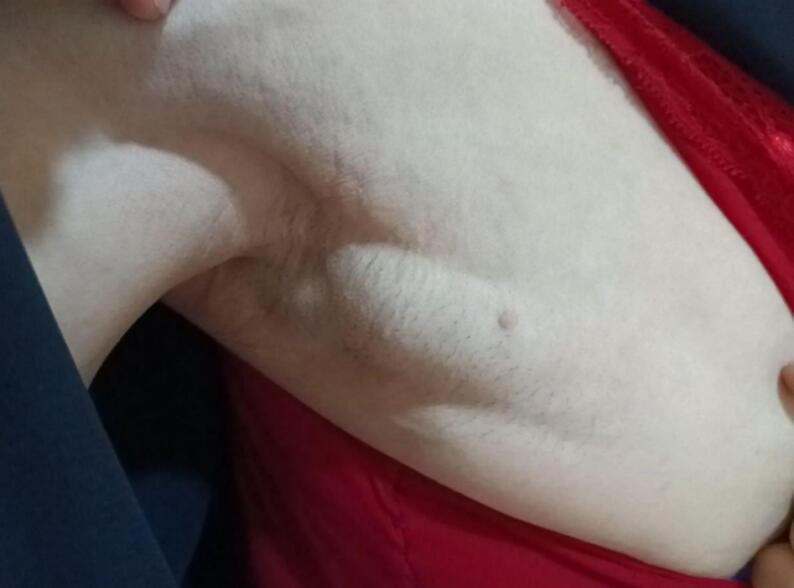
Emergence of lumps under both armpits and along the milk line, discharging a white fluid resembling milk.

**Fig. 4:**
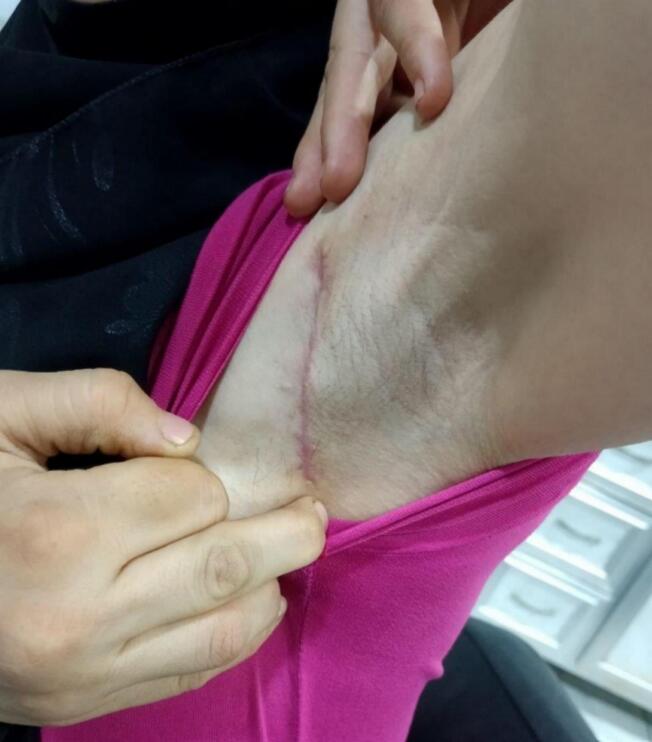
A photo for The patient after surgical excision of the accessory breast tissue.

A 34-year-old female presented to the dermatology department with concerns regarding significant facial changes and worsening wrinkles over the past five years, which have altered her facial features to the extent that she no longer recognizes herself Fig. 1. The patient reported difficulties in social interactions and noted an increase in adipose tissue in the abdominal and lower limb regions, accompanied by a reduction in adipose tissue in the upper body (chest and upper limbs) Fig. 2. Additionally, she complained of the emergence of lumps under both armpits and along the milk line, from which a white fluid (milk) was discharged Fig. 3. Her medical history was unremarkable, and she reported no current medications. Clinical examination revealed prominent cheekbones and hollowing around the eyes, with notable hypertrophy of the zygomatic and oral tuberculate muscles. Hematological tests indicated a low hemoglobin level of 11.30 g/dL (normal range: 12.0–15.5 g/dL) and a low complement component C3 level of 15 mg/dL (normal range: 90–180 mg/dL). Additionally, creatinine levels were low at 0.56 mg/dL (normal range: 0.6–1.2 mg/dL), and HDL cholesterol was low at 30 mg/dL (normal range: >40 mg/dL). Further evaluation of the breast tissue revealed glandular tissue consistent with accessory breast tissue bilaterally. An abdominal ultrasound was performed, which returned normal results. The patient was scheduled for surgical excision of the accessory breast tissue, which was successfully completed Fig. 4, and she was discharged from the hospital in good health.

## 3 Discussion

Acquired generalized lipodystrophy has been documented in over 250 patients, although it is likely under-recognized or underreported. Typically, patients present during their adolescent years. Recent suggestions aim to classify these individuals into three distinct categories: panniculitis, autoimmune, and idiopathic. Patients may either have one of these pre-existing conditions prior to the onset of generalized lipodystrophy or experience its spontaneous development. The condition affects women more frequently than men, with a ratio of approximately 3:1 [5].

It is characterized by a gradual, symmetrical loss of subcutaneous fat primarily from the face, neck, upper extremities, thorax, and abdomen, while sparing the lower extremities. Patients often exhibit a progeroid facial appearance, including complete loss of facial fat, a pinched nose, prominent upper incisors, and sunken eyes. The upper extremities may show reduced subcutaneous fat with prominent veins and muscles. Associated health issues may include mesangiocapillary glomerulonephritis and autoimmune diseases, such as systemic lupus erythematosus. Notably, there is usually no family history of lipodystrophy, and genetic mutations have not been consistently identified in affected individuals, indicating an unclear etiology. Regular monitoring is essential to prevent potential complications [6].

In patients presenting with symptoms suggestive of a lipodystrophy syndrome, a thorough metabolic workup is essential. This should include a complete metabolic panel to assess for hyperglycemia and any changes in hepatic enzyme levels. Additionally, a cholesterol panel should be conducted to exclude hypertriglyceridemia. Leptin levels can also be measured; low levels may indicate a potential response to replacement therapy. While genetic testing is not routinely performed, it may be considered for specific subtypes of lipodystrophy in clinical laboratories [7].

The differential diagnosis for severe weight loss should encompass conditions such as malnutrition, anorexia nervosa, uncontrolled diabetes mellitus, thyrotoxicosis, adrenocortical insufficiency, cancer cachexia, HIV-associated wasting, and chronic infections. Distinguishing lipodystrophy from uncontrolled diabetes can be particularly challenging, as both conditions may present with significant hypertriglyceridemia. However, achieving glycemic control in patients without lipodystrophy typically results in a restoration of body fat. Generalized lipodystrophies can be mistaken for insulin receptor mutations or acromegaly/gigantism, while familial partial lipodystrophy (FPLD) may be confused with Cushing's syndrome, truncal obesity, and multiple symmetric lipomatosis [8].

In our case, the 34-year-old female exhibited significant facial changes and worsening wrinkles over five years, similar to the progeroid appearance seen in Barraquer-Simons syndrome. She experienced increased adipose tissue in the abdomen and lower limbs, with corresponding fat loss in the upper body, reflecting the characteristic fat distribution of the syndrome.

Additionally, she presented with lumps under the armpits and discharge of a white fluid, indicating accessory breast tissue, which is not typical for Barraquer-Simons syndrome. Hematological tests showed low hemoglobin and complement component C3 levels, suggesting potential metabolic or immune issues.

While there are overlapping features, the presence of accessory breast tissue and specific lab findings make her case distinct from classic Barraquer-Simons syndrome.

Hematological tests showed low hemoglobin and complement component C3 levels, suggesting metabolic issues. The presence of accessory breast tissue further complicates her condition. Overall, the diagnostic approach, including clinical evaluation and surgical intervention for the accessory breast tissue, reflects standard practices for managing suspected lipodystrophy syndromes.

Management of lipodystrophies varies based on the specific subtype and the severity of associated metabolic abnormalities. For patients with significant metabolic issues, lipid-lowering agents and diabetic medications are crucial. Pioglitazone is preferred over metformin for increasing insulin sensitivity, and severe cases may necessitate insulin therapy. Leptin analogs, particularly metreleptin, are effective for patients who cannot produce leptin naturally and are the only FDA-approved replacement therapy for certain lipodystrophy cases. While hypertriglyceridemia may respond to troglitazone, interventions often have limited effectiveness for dyslipidemias. Additionally, cosmetic treatments like fillers (e.g., poly-L lactic acid and calcium hydroxyapatite) can be used for patients with lipodystrophy in sensitive areas. More research is needed to establish comprehensive diagnostic and management guidelines, with the expectation that further treatment options will develop as our understanding of metabolic regulation improves [9,10].

## 4 Conclusion

This case highlights the complexities of acquired partial lipodystrophy, particularly in a 34-year-old female presenting with unique features such as accessory breast tissue. The challenges in diagnosis and management underscore the importance of a thorough clinical evaluation and tailored therapeutic approaches. As our understanding of lipodystrophies evolves, ongoing research is essential to develop comprehensive diagnostic criteria and effective management strategies. This case not only contributes to the existing literature but also emphasizes the need for heightened awareness among healthcare providers to ensure timely diagnosis and intervention for patients with lipodystrophy syndromes.

## Author contribution

The work's conception and design: Mohammad Al-Jawad.

Paper writing, and article revision: Mohammad Al-Jawad, Lina Mawaldi, Nada Mawaldi.

Final revision and approval: Silva Ishkanian, Mariam Krimsty.

## Informed consent

Written informed consent was obtained from the patient for publication and any accompanying images. A copy of the written consent is available for review by the Editor-in-Chief of this journal on request.

## Consent for publication

All authors provide consent for publication.

## Ethical approval

This case report does not require ethical approval in our institution (University of Aleppo) as it involves a single patient case that is anonymized and does not include any identifiable personal information. The patient provided informed consent for the publication of this report.

## Guarantor

Silva Ishkanian.

## Research registration number

Our research study does not involve human subjects.

## Provenance and peer review

Not commissioned, externally peer-reviewed.

## Funding

There are no funding sources.

## Conflict of interest statement

The authors declare that they have no competing interests.
